# SIRT2 Inhibition Rescues Neurodegenerative Pathology but Increases Systemic Inflammation in a Transgenic Mouse Model of Alzheimer’s Disease

**DOI:** 10.1007/s11481-023-10084-9

**Published:** 2023-09-12

**Authors:** Noemi Sola-Sevilla, Alberto Mesa-Lombardo, Mikel Aleixo, Sara Expósito, Teresa Diaz-Perdigón, Amaya Azqueta, Farzad Zamani, Takayoshi Suzuki, Silvia Maioli, Francesca Eroli, Anna Matton, Maria J. Ramírez, Maite Solas, Rosa M. Tordera, Eduardo D. Martín, Elena Puerta

**Affiliations:** 1 Department of Pharmacology and Toxicology, University of Navarra, Navarra Institute for Health Research (IdiSNA), C/ Irunlarrea, 1, 31008 Pamplona, Spain; 2 Department of Anatomy, Histology and Neurosciences, Medical School, Autonoma University of Madrid, 28029 Madrid, Spain; 3 Laboratory of Neurophysiology and Synaptic Plasticity, Instituto Cajal, Consejo Superior de Investigaciones Científicas, Madrid, Spain; 4 SANKEN, Osaka University, Ibaraki, Osaka Japan; 5 Department of Neurobiology, Care Sciences and Society, Division of Neurogeriatrics, Center for Alzheimer Research, Karolinska Institutet, Stockholm, Sweden

**Keywords:** Alzheimer’s disease, Inflammation, Neurodegenerative diseases, Neuroinflammation, Sirtuin 2

## Abstract

Sirtuin 2 (SIRT2) has been proposed to have a central role on aging, inflammation, cancer and neurodegenerative diseases; however, its specific function remains controversial. Recent studies propose SIRT2 pharmacological inhibition as a therapeutic strategy for several neurodegenerative diseases including Alzheimer’s disease (AD). Surprisingly, none of these published studies regarding the potential interest of SIRT2 inhibition has assessed the peripheral adverse side consequences of this treatment. In this study, we demonstrate that the specific SIRT2 inhibitor, the compound 33i, does not exhibit genotoxic or mutagenic properties. Moreover, pharmacological treatment with 33i, improved cognitive dysfunction and long-term potentiation, reducing amyloid pathology and neuroinflammation in the APP/PS1 AD mouse model. However, this treatment increased peripheral levels of the inflammatory cytokines IL-1β, TNF, IL-6 and MCP-1. Accordingly, peripheral SIRT2 inhibition with the blood brain barrier impermeable compound AGK-2, worsened the cognitive capacities and increased systemic inflammation. The analysis of human samples revealed that SIRT2 is increased in the brain but not in the serum of AD patients. These results suggest that, although SIRT2 pharmacological inhibition may have beneficial consequences in neurodegenerative diseases, its pharmacological inhibition at the periphery would not be recommended and the systemic adverse side effects should be considered. This information is essential to maximize the therapeutic potential of SIRT2 inhibition not only for AD but also for other neurodegenerative diseases.

**Graphical Abstract:**

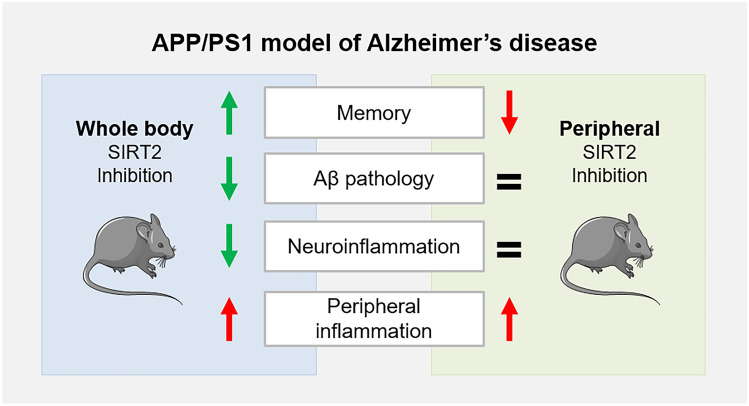

**Supplementary Information:**

The online version contains supplementary material available at 10.1007/s11481-023-10084-9.

## Introduction

Sirtuin 2 (SIRT2) is one of the seven members of the sirtuin family, a highly conserved NAD^+^-dependent histone deacetylases (HDACs), that target histone and non-histone substrates. Specifically, SIRT2 shows some characteristics that make it special compared to the rest of the sirtuins and that have caused the interest in this enzyme to grow exponentially in recent years. Firstly, it is the sirtuin with the highest expression in the brain, although it is also expressed in a wide range of tissues and organs including the adipose tissue, muscle, liver, testes, heart, kidney and macrophages (North et al. 2014; Maxwell et al. 2011). Moreover, within the cell, it is the only member of the family mainly located in the cytoplasm (Jayasena et al. 2016) with the ability to translocate to the nucleus (North et al. 2003) and it is also found in the mitochondria (Liu et al. 2017). Its ubiquitous distribution supports the wide variety of substrates deacetylated by SIRT2 and its participation in multiple cellular processes including cytoskeletal stabilization, DNA repair, gene transcription, autophagy, myelin formation and inflammation (for a review, see Wang et al. 2019). All these characteristics have made SIRT2 an interesting pharmacological target. However, the consequences of its enzymatic modulation have not always been easy to predict, which raises the question about the safety of treatments aimed at modulating SIRT2 activity.

Although it has been proposed that SIRT2 has a central role on several pathological conditions, its specific function remains controversial (Chen et al. 2021). For instance, when it comes to deciphering the role of SIRT2 on inflammation, different researchers support that it can either prevent (Pais et al. 2013; Rothgiesser et al. 2010; Yuan et al. 2016; Zhang and Chi 2018; Lo Sasso et al. 2014) or promote it (Lee et al. 2014; Chen et al. 2015; Jiao et al. 2020; Wang et al. 2016; Wu et al. 2020). In the same line, in recent years, a growing body of evidence has proposed a role for SIRT2 in tumorigenesis, however, its role in cancer is complicated as it has been described as both an oncogene and a tumor suppressor (for a review see Zhang et al. 2020). In addition, SIRT2 presents several isoforms and polymorphisms (Rack et al. 2014; Maxwell et al. 2011; Shen et al. 2020), which may show different sensitivity or affinity for the different pharmacological inhibitors, complicating the interpretation of all these studies.

The role of SIRT2 on aging is also a matter of debate. Several studies have shown age-related changes in SIRT2 both in the periphery and in the central nervous system (CNS), however they show contradictory data (for a review, see Sola-Sevilla et al. 2021). Some authors have described that SIRT2 is decreased in aged-hematopoietic stem cells (Chambers et al. 2007; Luo et al. 2019), in blood mononuclear cells (Yudoh et al. 2015) and in macrophages isolated from old mice (He et al. 2020). Moreover, He et al. (2020) suggest that peripheral SIRT2 is essential to prevent aging-associated inflammation and insulin resistance (He et al. 2020). Accordingly, overexpression of SIRT2 enhanced median lifespan in mice suggesting the potential of SIRT2 to delay aging and age-related diseases (North et al. 2014). However, others have shown increased levels of SIRT2 with aging in the CNS (Maxwell et al. 2011) suggesting that SIRT2 is deleterious and promotes neurodegeneration (Diaz-Perdigon et al. 2020). Notably, we have recently demonstrated that the overexpression of isoform 3 of SIRT2 increases neuroinflammation in a mouse model of aging but not in a control strain, confirming that in combination with other risks factors, increased levels of SIRT2 in the CNS could be detrimental (Sola-Sevilla et al. 2021). Overall, all these studies support the notion that age-related changes in SIRT2 and the consequences of its modulation are dependent on the cell type and the context, and differ significantly between the CNS and the periphery.

In this scenario, there are several studies demonstrating that SIRT2 pharmacological inhibition provides beneficial effects in different animal models of aging (Diaz-Perdigon et al. 2020), depression (Guclu et al. 2022; Muñoz-Cobo et al. 2017; Erburu et al. 2017), Huntington´s (Luthi-Carter et al. 2010; Chopra et al. 2012), Parkinson's (Esteves et al. 2019; Sun et al. 2018; de Oliveira et al. 2017) and Alzheimer’s disease (AD) (Bai et al. 2022; Wang et al. 2020; Biella et al. 2016), pointing out the potential role of SIRT2 as a therapeutic target for these neurodegenerative diseases. However, despite the aforementioned controversy between potential beneficial or detrimental effects of SIRT2 modulation, none of these studies have addressed the peripheral side effects of SIRT2 inhibition in these models.

The general objective of this study is to evaluate the behavioral and molecular effects that the treatment with a specific SIRT2 inhibitor, the compound 33i, has in a transgenic mouse model of AD, the APP/PS1 model, both at central and peripheral levels. Specifically, after confirming its safety in the in vitro toxicological studies, the effect of 33i on AD-associated symptoms such as anxiety, learning and memory dysfunction, amyloid pathology and neuroinflammation will be assessed. These determinations will be accompanied by a metabolic characterization (glucose and insulin-tolerance tests) and the evaluation of peripheral inflammation. This will allow us to evaluate if the pharmacological inhibition of SIRT2 is an effective and safe strategy, and if its usefulness for the treatment of neurodegenerative diseases can be considered.

## Results

### In Vitro Toxicological Studies with the Compound 33i

Regarding the potential usefulness of SIRT2 inhibitors as pharmacological treatments for neurodegenerative diseases, the implication of HDACs in gene expression and the associated potential for DNA toxicity upon their inhibition present a major concern. Therefore, we firstly performed a preliminary toxicity evaluation of 33i in vitro, essential for translational proposals. Specifically, the Ames test was used to study gene mutations, survival and proliferation was measured by counting the cells 3 and 48 h after treatment respectively, and cytotoxicity was assessed with the MTT test. Finally, modified-comet assays were performed to detect DNA strand breaks (SBs), alkali-labile sites (ALS) and oxidized bases.

**Fig. 1:**
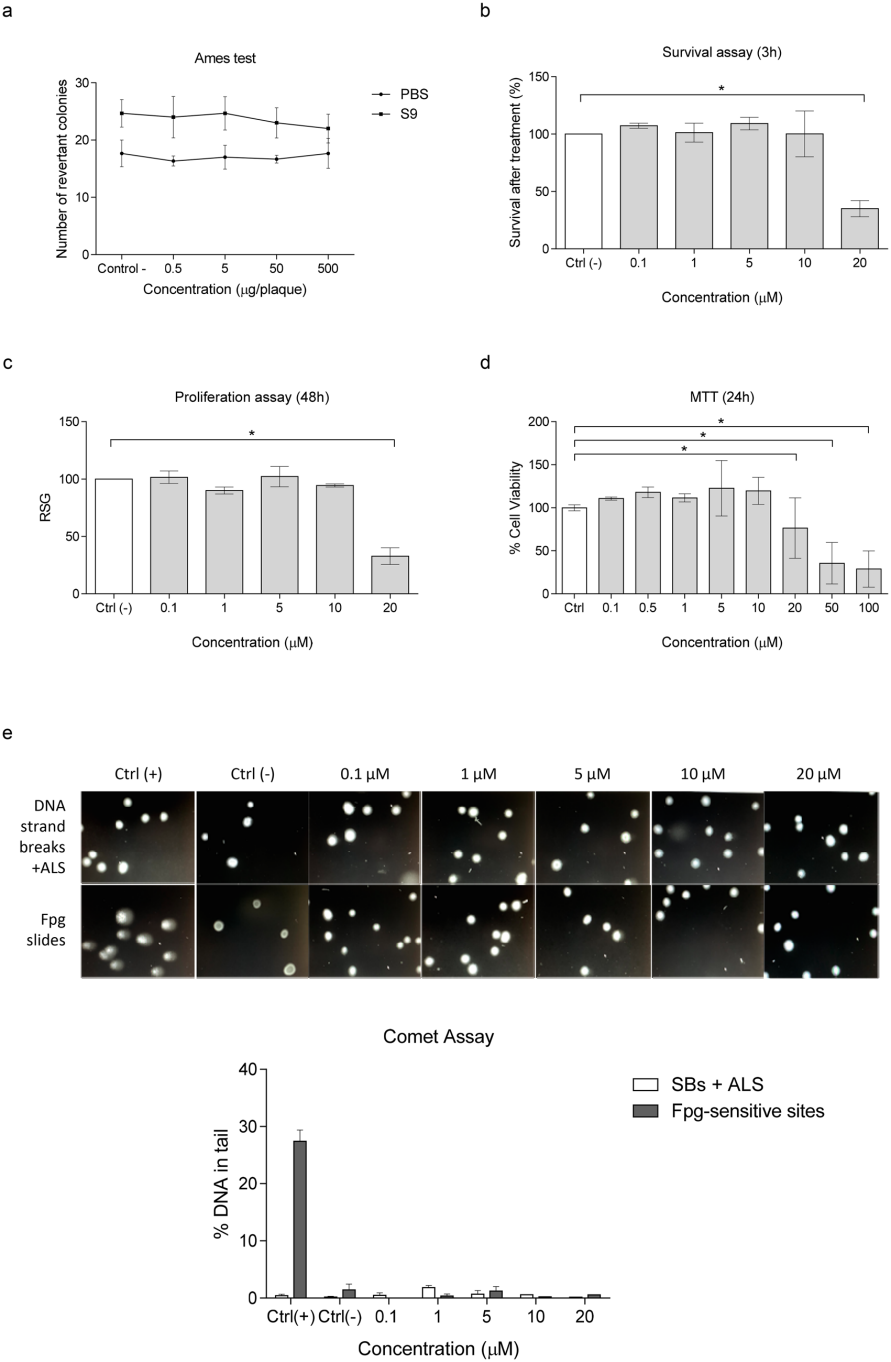
In vitro toxicological evaluation of 33i compound. (**a**) The results obtained from the Ames test revealed no mutagenicity caused by 33i or its metabolites. Results are presented as number of revertant colonies. (**b**) and (**c**) Effect of different 33i concentrations on the survival and proliferation of SH-SY5Y 3 h and 48 h after 3 h-treatment. Results are presented as survival after treatment (%) (**b**) and Relative Suspension Growth (RSG) (%) (**c**) (*p < 0.05, one-way ANOVA followed by Dunnett's multiple comparisons test). (**d**) Cytotoxicity of the 33i on SH-SY5Y cells using the MTT assay; SH-SY5Y cells were incubated with 33i for 24 h (*p < 0.05, one-way ANOVA followed by Dunnett's multiple comparisons test). (**e**) Genotoxic evaluation of the compound 33i on SH-SY5Y cells using the standard and Fpg-modified comet assays. Representative images (top) and quantitative measurement (bottom) of the effect of different concentrations of 33i on DNA strand breaks and net Fpg-sensitive sites in SH-SY5Y cell line. Cells treated with 20 µM MMS were used as positive control. Results are presented as % tail DNA. All the results are expressed as mean ± SEM

As shown in Fig. 1a, 33i did not induce a dose-dependent increase of *Salmonella typhimurium* TA98 revertant colonies in the presence or absence of metabolic activation (i.e., S9 fraction). Noteworthy, the number of revertant colonies in the positive controls with and without metabolic activations showed the expected results (10 µg/plaque 2-aminofluorene + S9: 2633.3 ± 21; 20 µg/plaque 4-nitro-o-phenylenediamine: 1794.7 ± 16.2) supporting the validity of the obtained results. These results show that 33i, or its metabolites, does not induce point mutations.

Before performing the comet assay in a neuroblastoma cell line (SH-SY5Y cells), the cytotoxicity was measured as suggested by Azqueta et al (2022). In this regard, the cytotoxicity of 33i was studied after 3 h of treatment by counting the cells after washing them (survival) and performing the proliferation assays. As seen in Fig. 1b, c, 20 µM of 33i showed an effect reducing the survival in SH-SY5Y cell line. However, lower concentrations of 33i did not show changes in the rate of survival or cell proliferation. The cytotoxicity of 33i was also studied using the MTT assay after 24 h; results showed that 20 µM decreased the percentage of survival while the lower concentrations tested were not cytotoxic (Fig. 1d).

As seen in Fig. 1e, no effect was observed in the percentage of tail DNA in cells treated with different concentrations of 33i (a toxic and several non-toxic ones), suggesting that it did not produce SBs or ALS. Moreover, 33i did not induce an increase in the Fpg-sensitive sites detected after a 3 h treatment. These results indicate that 33i does not produce DNA oxidation (or methylation) in the 8-oxoguanine bases of DNA at the concentrations tested.

### 33i Treatment Ameliorates Cognitive Performance in APP/PS1 Mice

**Fig. 2:**
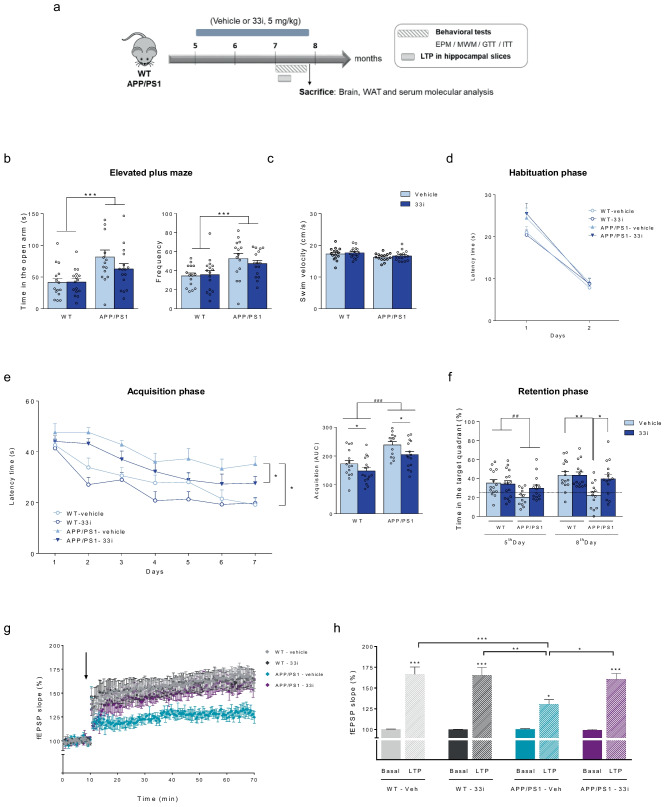
33i treatment improves learning and memory in APP/PS1 mouse model. (**a**) The experimental paradigm. Five-month-old male and female wild-type (WT) and APP/PS1 mice were treated daily with 33i (5 mg/kg i.p.) or vehicle (see Materials and methods) for 3 months (WT-vehicle n = 15; WT-33i n = 16; APP/PS1-vehicle n = 14; APP/PS1-33i n = 15 animals). Body weight of the mice was recorded weekly during the period of drug administration. Behavioral tests started at the beginning of the 3^rd^ month and during these days, the treatment was given at the end of the behavioral test. The behavioral tests included the elevated plus maze (EPM) and the Morris water maze (MWM). Glucose homeostasis was also assessed by the glucose and insulin-tolerance tests (GTT and ITT respectively). (**b**) Time in open arms (left) (F = 14.47) and quantity of times that mice enter in the open arms from close arms (right) (F = 13.20) were analyzed in the EPM test (***p < 0.001, main effect of genotype, two-way ANOVA). (**c**) Swim velocity measured during the first trial of the habituation phase of the MWM. Note that no significant differences were detected among all four groups. (**d**) Habituation phase of the MWM. (**e**) Escape latency in the acquisition phase of the MWM. Note that APP/PS1 mice had significant higher escape latency than WT mice, an effect partially reversed by 33i treatment (*p < 0.05, two-way Repeated Measures ANOVA). (Right) Area under the curve (AUC) of the acquisition curve (F = 6.310, *p < 0.05 main effect of treatment; F = 27.34, ###p < 0.001 main effect of genotype, two-way ANOVA). (**f**) Representation of the percentage of time spent in the correct quadrant in the retention phase of the MWM (5^th^ Day: F = 8.382 ##p < 0.01, main effect of genotype; 8^th^ Day: F = 5.657, *p < 0.05; **p < 0.01, two-way ANOVA followed by Tukey’s test). (**g**) Time course of mean fEPSP slope in vehicle and 33i-treated WT and APP/PS1 mice hippocampal slices. Arrow corresponds to theta burst stimulation (TBS). (**h**) Average relative changes of fPSP slope before TBS (basal) and 60 min after TBS in vehicle and 33i treated WT and APP/PS1 mice (n = 4 animals per group, 3–4 slices per animal; F = 31.08, *p < 0.05, **p < 0.01, ***p < 0.001, one-way ANOVA). In all figures results are expressed as mean ± SEM

To assess if SIRT2 inhibition could delay the pathology of the disease, 5 months-old wild-type (WT) and APP/PS1 mice were treated with 33i (5 mg/kg) for 3 months. Behavioral tests started at the beginning of the 3rd month and, during these days, the treatment was given at the end of the behavioural test (Fig. 2a).

We first performed the Elevated Plus Maze test (EPM) to assess the anxiety-like behavior of the animals, an aspect known to be altered in AD transgenic mouse models (Olensen et al. 2016) as well as in patients (Eratne et al. 2018). As it can be seen in Fig. 2b, APP/PS1 mice spent significantly more time in the open arms of the maze and showed a higher frequency in entering to the open arms when compared to WT mice. This suggests an increased risk-taking behavior and a decreased anxiety-like behavior, not affected by the treatment (Fig. 2b).

Then, mice were subjected to the Morris Water Maze (MWM) task to assess the effect of 33i treatment on spatial learning and memory. On the habituation phase, no differences were found when swimming speed of the animals was analyzed (Fig. 2c). Moreover, it was confirmed that all mice exhibited a normal swimming pattern and were able to reach the visible platform since no significant differences were observed in this phase among all four groups (Fig. 2d). These results enabled us to exclude the effect of motivational and sensorimotor factors on animal learning and memory performance. In the acquisition phase, the time spent to find the platform was significantly reduced each day in WT mice whereas it was significantly higher in APP/PS1 mice confirming that this strain shows learning and memory deficits at this age (Fig. 2e). Interestingly, 33i-treated animals showed a better performance in this test compared with their corresponding vehicle-treated groups (Fig. 2e). This suggests that 33i-treatment improved learning capacity in the WT group and reversed learning deficits in APP/PS1 mice.

On 5th and 8th day a probe trial was performed to evaluate the memory retention, which is represented as the percentage of time spent swimming in the target quadrant for 60 s. As shown in Fig. 2f, the first day of the probe trial (5^th^ day), APP/PS1 mice spent less than the 25% of the total trial duration in the target quadrant indicating that these mice show cognitive deficiencies. No significant differences were observed between vehicle and 33i-treated animals. However, the probe trial of 8th day revealed that, while vehicle-treated APP/PS1 animals still did not remember the location of the platform, 33i-treated APP/PS1 mice showed good memory retention with a percentage time in the correct quadrant similar to WT mice.

We also evaluated the effect of 33i treatment on synaptic plasticity by assessing long-term potentiation (LTP) in ex vivo hippocampal slices from WT and APP/PS1 mice. As shown in Fig. 2g, h, LTP in vehicle-treated APP/PS1 mice was substantially reduced compared to that observed in slices from WT mice. In concordance with the cognitive improvement observed in the MWM, 33i treatment restored LTP in the APP/PS1 slices to the level seen in the WT slices.

### 33i Treatment Reduces Amyloid Pathology and Neuroinflammation

**Fig. 3:**
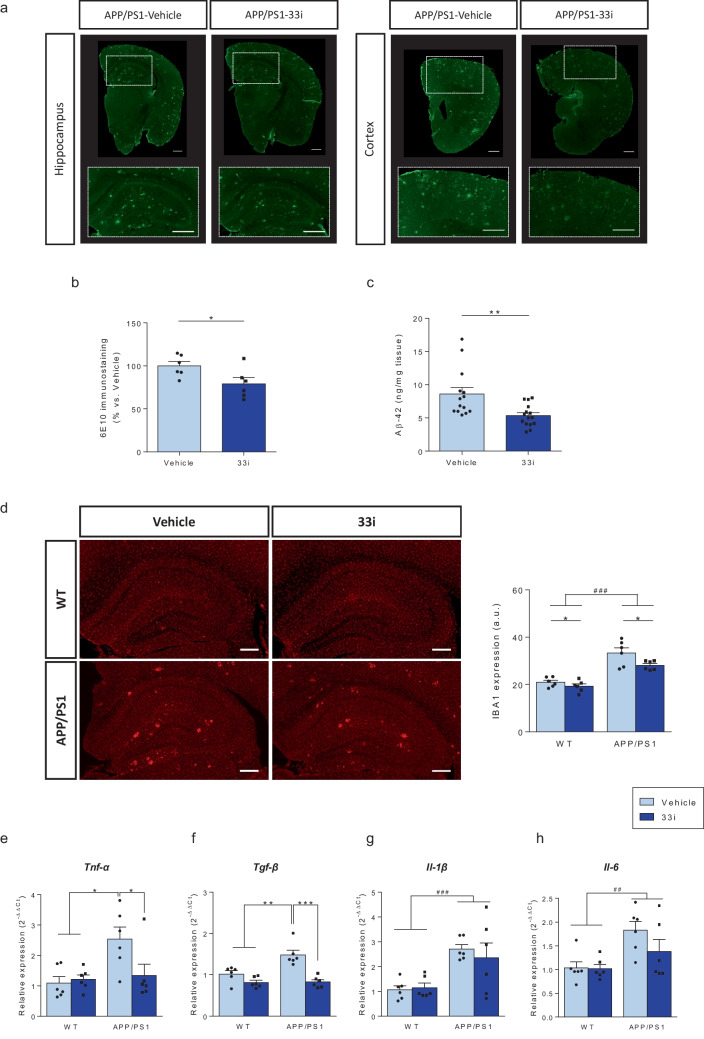
33i treatment reduces amyloid pathology and neuroinflammation in APP/PS1 mice. (**a**) Representative hippocampal and cortical sections of β-amyloid plaques stained with 6E10 antibody in brain slices of 8 months-old APP/PS1 mice treated with vehicle or 33i. Amyloid deposits were absent in age-matched WT mice (data not shown). Scale bar = 500 µm. (**b**) Amyloid burden quantification (n = 6 mice per group, 2 sections including hippocampus and frontal cortex per animal; *p < 0.05, Student’s t-test). (**c**) Levels of Aβ-42 in the cortex of vehicle and 33i treated-APP/PS1 mice measured by ELISA (n = 14–15 mice per group, **p < 0.01, Student’s t-test). (**d**) Representative immunofluorescence images (left) and quantitative measurement (right) of Iba-1 expression (n = 6 animals per group, 2 sections including hippocampus and cortex per animal) (F = 7.216, *p < 0.05 main effect of treatment; F = 65.00, ###p < 0.001 main effect of genotype, two-way ANOVA). Scale bar = 250 µm. Hippocampal gene expression of (**e**) *Tnf-α* (F = 4.864, *p < 0.05, two-way ANOVA followed by Tukey’s test), (**f**) *Tgf-β* (F = 8.336, **p < 0.01 and ***p < 0.001, two-way ANOVA followed by Tukey’s test), (**g**) *Il-1β* (F = 17.73, ###p < 0.001, main effect of genotype, two-way ANOVA) and (**h**) *Il-6* (F = 10.98, ##p < 0.01, main effect of genotype, two-way ANOVA). *Gapdh* was used as an internal control (n = 6 animals per group). In all panels, results are shown as mean ± SEM

We next evaluated the effect of 33i on Aβ pathology, one of the main neuropathological hallmarks of AD. Interestingly, 33i treatment significantly reduced Aβ burden, evidenced as a significant reduction in 6E10 immunostaining (Fig. 3a, b) and Aβ-42 levels (Fig. 3c) in APP/PS1 mice (no Aβ was detected in non-transgenic WT littermates, data not shown). The reduction in amyloid pathology in the cortex and hippocampus was accompanied by a significant decrease in different neuroinflammatory markers such as the microglial Iba-1 (Fig. 3d) and the gene expression of inflammatory cytokines *Tnf-α* (Fig. 3e) and *Tgf-β* (Fig. 3f). Regarding *Il-1β* and *Il-6* gene expression, a tendency towards lower levels was observed in 33i-APP/PS1 group; however, statistical analysis revealed only a significant main effect of genotype (Fig. 3g, h).

### 33i-Treated APP/PS1 Mice Exhibit Increased Microglial Aβ Phagocytosis

**Fig. 4:**
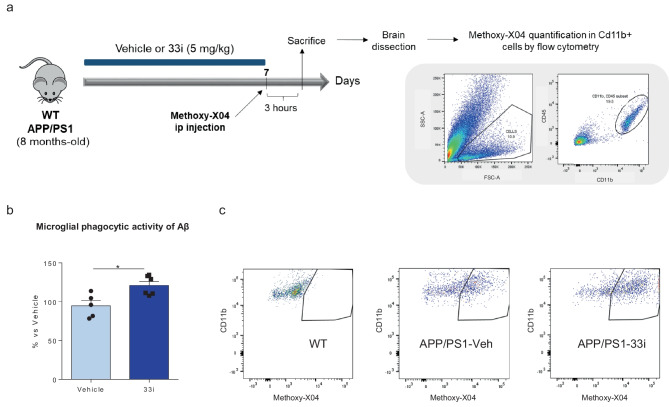
SIRT2 inhibition increases microglial phagocytosis of methoxy-labeled Aβ. (**a**) Experimental design for quantitative in vivo assessment of amyloid-beta phagocytic capacity and gating strategy to identify CD11b^+^CD45^low^ microglia. SSC: side scatter; FSC: forward scatter. (**b**) Quantification of Aβ phagocytosis by flow cytometry of microglia isolated from vehicle or 33i treated 8 months-old APP/PS1 mice 3 h after intraperitoneal injection of methoxy-X04 (*p < 0.05, Student’s t-test). Results are shown as mean ± SEM (n = 5–6 animals per group). (**c**) Representative FACS plots demonstrating the engulfment of Aβ by microglia isolated from APP/PS1 mice upon treatment with vehicle or 33i. Wild-type mice (WT) injected with methoxy-X04 were used to determine the methoxy-X04-threshold for non-phagocytic cells

It has been described that, in early stage of AD, microglial activation delays disease progression by promoting clearance of Aβ by phagocytosis (Park et al. 2019; Yamanaka et al. 2012). Interestingly, a recent study has demonstrated that SIRT2 deficiency enhances bacterial phagocytosis by macrophages (Ciarlo et al. 2017). Thus, in order to provide a plausible mechanism underlying the reduction in amyloid pathology after 33i treatment, mice were treated with methoxy-X04 (a fluorescent derivative of Congo red, which crosses the blood–brain barrier (BBB) and has high Aβ-binding affinity). Adult microglial cells were isolated and analyzed for methoxy-X04 fluorescence by flow cytometry (Fig. 4a). As shown in Fig. 4b, c a significant increased signal of methoxy-X04-labeled Aβ was observed in microglia of 33i-treated APP/PS1 mice compared to vehicle treated mice indicating that 33i treatment increases microglial Aβ phagocytosis in vivo. Supporting the validity and the specificity of the obtained results, no methoxy-X04 signal was observed in WT animals (Fig. 4c).

### 33i Treatment Induces Systemic Inflammation

**Fig. 5:**
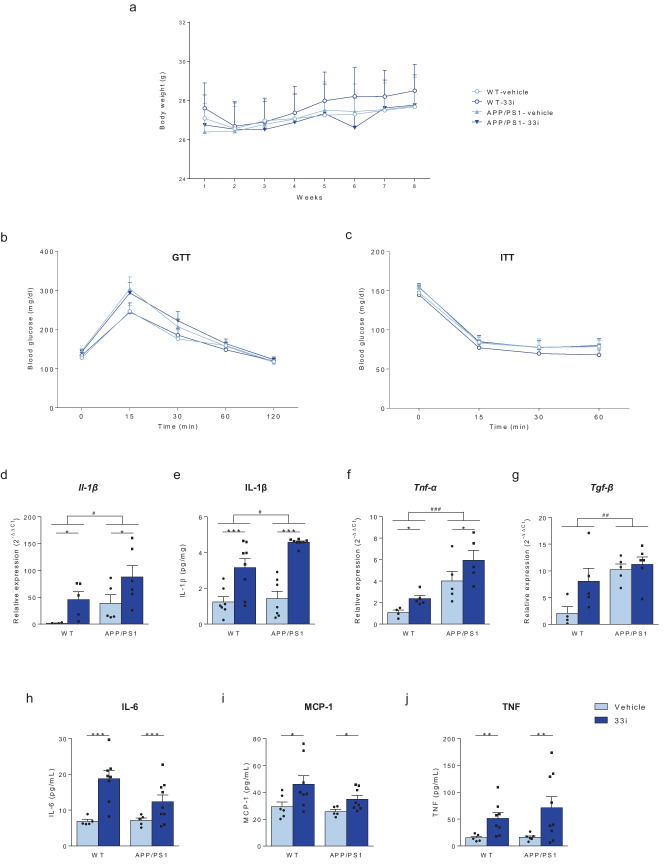
SIRT2 inhibition induces peripheral inflammation. (**a**) Weekly body weight monitoring of WT and APP/PS1 mice during the treatment. Glucose (**b**) and Insulin (**c**) tolerance tests. 33i treatment for two months in WT and APP/PS1 mice did not have any significant effect on glucose and insulin tolerance (n = 12–14 animals per group). (**d**) Gene expression of *Il-1β* (F = 7.529, *p < 0.05, main effect of treatment; F = 5.532, #p < 0.05, main effect of genotype, two-way ANOVA, n = 5–6 mice per group) and (**e**) protein expression of IL-1β (F = 50.13, ***p < 0.01, main effect of treatment; F = 4.978, #p < 0.05, main effect of genotype, two-way ANOVA, n = 7–8 animals per group) in white adipose tissue of WT and APP/PS1 mice. Note that 33i treatment increased levels of this pro-inflammatory cytokine in WT and APP/PS1 animals. Peripheral gene expression of (**f**) *Tnf-α* (F = 5.201, *p < 0.05, main effect of treatment; F = 21.11, ###p < 0.001, main effect of genotype, two-way ANOVA) and (**g**) *Tgf-β* (F = 11.46, ##p < 0.01, main effect of genotype, two-way ANOVA) (n = 5–6 animals per group). *36b4* was used as an internal control. Serum levels of the cytokines (**h**) IL-6 (F = 18.76, ***p < 0.001, main effect of treatment, two-way ANOVA), (**i**) MCP-1 (F = 7.782, *p < 0.05, main effect of treatment, two-way ANOVA) and (**j**) TNF (F = 8.901, **p < 0.01, main effect of treatment, two-way ANOVA) (n = 5–8 mice per group). Results are shown as mean ± SEM

A recent study has demonstrated that SIRT2 is necessary and beneficial for preventing aging and overnutrition-associated chronic inflammation and insulin resistance (He et al. 2020). Thus, in order to evaluate possible peripheral adverse side effects derived from SIRT2 inhibition, we next assessed the systemic inflammation and metabolic homeostasis in 33i-treated animals. Body weight data were collected weekly throughout the study, as age, disease and treatment could alter body mass; however, no significant differences among experimental groups were observed (Fig. 5a). Moreover, not significant differences between vehicle or 33i-treated animals were observed when glucose (Fig. 5b) and insulin (Fig. 5c) tolerance were assessed. However, the molecular analysis of pro-inflammatory cytokines at the periphery revealed significantly increased levels of IL-1β (Fig. 5d, e) and *Tnf-α* (Fig. 5f). Regarding *Tgf-β*, a significant main effect of strain and a tendency towards higher levels in 33i-treated animals was found (Fig. 5g). Moreover, although no significant differences were observed in serum levels of some cytokines analyzed such as INF, IL-10 or IL-12p70 (data not shown), the protein level of IL-6 (Fig. 5h), MCP-1 (Fig. 5i) and TNF (Fig. 5j) was significantly higher in both WT and APP/PS1 treated with 33i compared to vehicle-treated animals.

### SIRT2 Peripheral Inhibition with AGK-2 Worsens Learning and Memory Capacities And Induces Systemic Inflammation

To confirm the deleterious effects derived from systemic SIRT2 inhibition, we next repeated the same experimental paradigm but with a SIRT2 inhibitor unable to cross the BBB, the compound AGK-2 (Fig. S1). It has been described that SIRT2^−/−^ mice and SIRT2 pharmacological inhibition induce hippocampal GluA1 accumulation (Wang et al. 2017; Diaz-Perdigón et al. 2020). As expected, 33i treatment significantly increased the hippocampal expression of GluA1 (Fig. S1a) while this effect was not observed after AGK-2 treatment, confirming its inability to cross the BBB (Fig. S1b). Both compounds inhibited SIRT2 enzyme at the peripheral level, evidenced by an increase in the gene expression of ATP-binding cassette transporter Abca1 (a known transporter of cholesterol whose transcription is inhibited by SIRT2) in white adipose tissue (Spires-Jones et al. 2012; Diaz-Perdigón et al. 2020) (Fig. S1c, d).

**Fig. 6:**
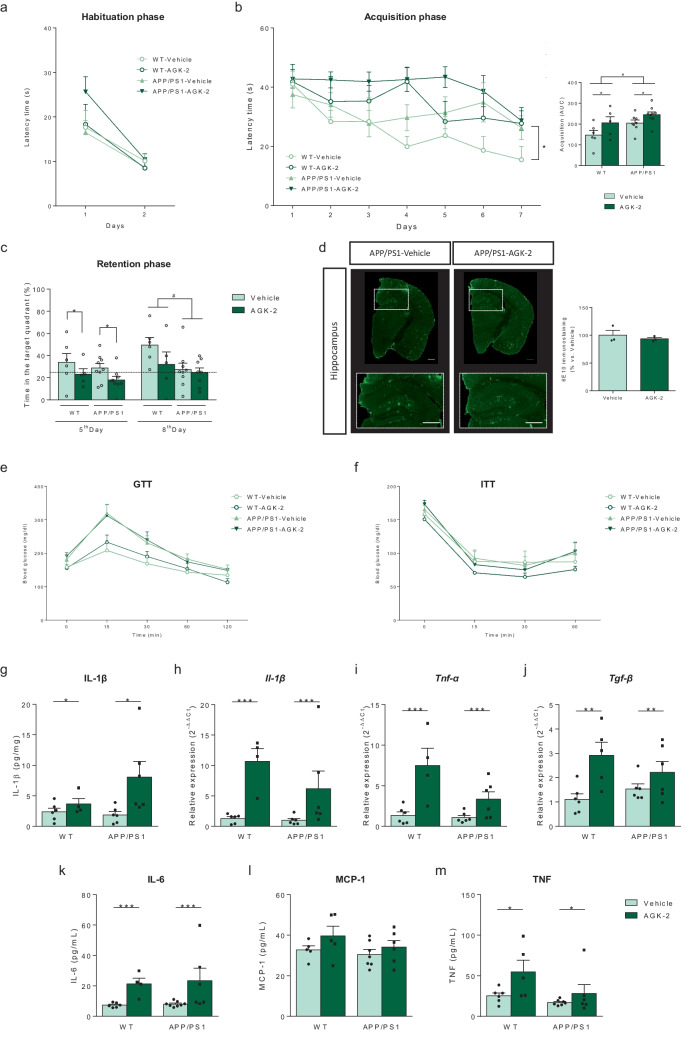
Peripheral SIRT2 inhibition impairs memory and increases systemic inflammation. (**a**) Habituation phase of the MWM. (**b**) Escape latency in the acquisition phase of the MWM and corresponding area under the curve (AUC) of the acquisition curve (F = 6.716, *p < 0.05 main effect of treatment; F = 6.580, #p < 0.05 main effect of genotype, two-way ANOVA, n = 6–8 animals per group). Note that AGK-2 treatment worsened learning capacities in both WT and APP/PS1 mice. (**c**) Representation of the percentage of time spent in the correct quadrant in the retention phase of the MWM (5^th^ Day: F = 4.474 *p < 0.05, main effect of treatment; 8^th^ Day: F = 4.854, #p < 0.05, main effect of genotype, two-way ANOVA). (**d**) Representative hippocampal sections of β-amyloid plaques stained with 6E10 antibody in brain slices (left) and amyloid burden quantification (right) in 8 months-old APP/PS1 mice treated for two months with vehicle or AGK-2 (n = 3 animals per group, 2 sections including hippocampus and frontal cortex per animal) Scale bar = 500 µm. Glucose (**e**) and Insulin (**f**) tolerance tests. No significant differences were observed between vehicle or AGK-2 treated animals (n = 5–9 mice per group). Peripheral protein expression of (**g**) IL-1β (F = 5.951, *p < 0.05, main effect of treatment, two-way ANOVA) and gene expression of (**h**) *Il-1β* (F = 16.33, ***p < 0.001, main effect of treatment, two-way ANOVA), (**i**) *Tnf-α* (F = 19.60, ***p < 0.001, main effect of treatment, two-way ANOVA) and (**j**) *Tgf-β* (F = 11.49, **p < 0.01, main effect of treatment, two-way ANOVA) (n = 6 animals per group). *36b4* was used as an internal control. Serum levels of the cytokines (**k**) IL-6 (F = 10.80, ***p < 0.001, main effect of treatment, two-way ANOVA), (**l**) MCP-1 and (**m**) TNF (F = 5.926, *p < 0.05, main effect of treatment, two-way ANOVA) (n = 5–8 mice per group). Results are shown as mean ± SEM

AGK-2 treatment did not induce any significant effect on body weight (data not shown), swim velocity (data not shown) or the habituation phase of the MWM (Fig. 6a). However, as seen on Fig. 6b, this treatment not only did not improve the cognitive decline observed in APP/PS1 at this age, but it also worsened the performance of both strains, WT and APP/PS1 in the acquisition phase of the MWM. This deleterious effect was further confirmed on the 5^th^ day of the retention phase where vehicle-treated animals were able to remember the location of the platform but AGK-2-treated animals did not (Fig. 6c). Regarding the neuropathological hallmarks observed in the hippocampus of APP/PS1 mice, AGK-2 treatment did not reduce neither β-amyloid burden (Fig. 6d) nor neuroinflammatory cytokines levels (Fig. S2). Moreover, although no significant differences were observed between vehicle and AGK-2 treated animals when glucose (Fig. 6e) and insulin (Fig. 6f) tolerance tests were performed, at the periphery AGK-2 treatment increased the expression of IL-1B (Fig. 6g, h), *Tnf-α* (Fig. 6i), *Tgf-β* (Fig. 6j), IL-6 (Fig. 6k), MCP-1 (Fig. 6l), and TNF (Fig. 6m).

### SIRT2 Is Upregulated in Postmortem Cerebral Cortex Samples from AD Patients

**Fig. 7:**
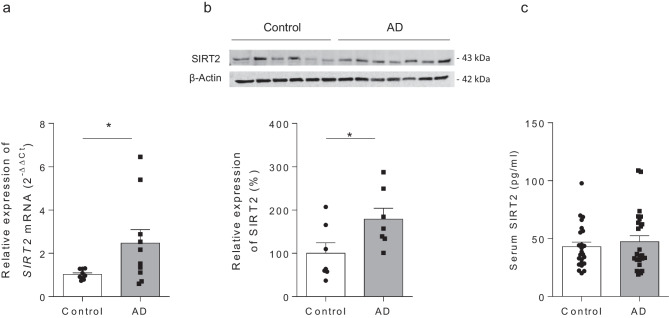
SIRT2 is increased in postmortem brain tissue from Alzheimer’s disease patients but not in serum. (**a**) Gene expression of *SIRT2* in frontal cortex of *postmortem* control and Alzheimer’s disease (AD) human samples (*p < 0.05, Student’s t-test). *β-ACTIN* was used as internal control (n = 10 samples per group). (**b**) Representative western blot images (top) and SIRT2 protein levels quantification (bottom) in frontal cortex of *postmortem* control and AD human samples (*p < 0.05, Student’s t-test). *β-ACTIN* was used as internal control (n = 7 samples per group). (**c**) No significant differences between both groups were found when SIRT2 was analysed in serum samples (n = 24 samples per group)

Given the different roles that SIRT2 seems to be playing at central and peripheral levels, we finally analyzed its expression in brain and plasma samples from AD patients. As shown in Fig. 7, *SIRT2* gene expression and protein levels are increased in the cerebral cortex (Fig. 7a, b) but remains unchanged in serum samples (Fig. 7c) from AD patients compared to control group.

## Discussion

Recent studies have proposed SIRT2 as a key player in aging, inflammation, cancer and neurodegenerative diseases, but its specific role in these processes seems contradictory. On the one hand, it has been demonstrated that SIRT2 knockout (SIRT2^−/−^) mice show aberrant synaptic plasticity together with impaired learning and memory (Wang et al. 2017). In line with this notion, it has been suggested that SIRT2 could be linked to several key processes in the control of aging process like caloric restriction and oxidative stress resistance (North et al. 2014). In addition, a recent study has shown that SIRT2 prevents and reverses age-related inflammation and insulin resistance (He et al. 2020). On the other hand, several studies suggest that SIRT2 is deleterious promoting neurodegeneration and have shown that its pharmacological inhibition provides beneficial effects in different neuropsychiatric and neurodegenerative diseases such as depression, Huntington’s disease, Parkinson’s disease and AD (for a review, see Sola-Sevilla et al. 2020; Sola-Sevilla and Puerta 2024). Based on these considerations, it seems reasonable to think that SIRT2 could have different functions, beneficial or detrimental, in diverse circumstances and environments (Chen et al. 2021). Thus, further studies are needed to fully understand the specific role of SIRT2 on different organs and functions, and to determine the safety and potential side effects of its pharmacological inhibition. This knowledge is an essential step to determine if SIRT2 could be postulated as a good pharmacological target.

HDAC inhibitors, in general, present a major concern because of their potential implication in mutagenic or genotoxic processes. In fact, HDAC inhibitors have been described as potential cytotoxic and genotoxic molecules although the underlying mechanisms remain unknown (Olaharski et al. 2006; Bose et al. 2014; Yoo and Lee 2005; Johnson and Walmsley 2013). Specifically, SIRT2 has been considered indispensable during carcinogenesis; however, there is now a significant controversy regarding whether SIRT2 is an oncogene or a tumor suppressor (Zhang et al. 2020). In this context, although numerous studies have proposed the use of SIRT2 inhibitors for different neurodegenerative diseases (for a review, see Sola-Sevilla et al. 2020), to date, none of them has analyzed the potential adverse side effects of this treatment. For this reason, we carried out a preliminary genotoxicological study with the potent and specific SIRT2 inhibitor 33i (Suzuki et al. 2012). The results obtained from the Ames test discard that 33i or its metabolites are mutagenic; however, further mutagenic studies should be carried out in other *Salmonella typhimurium* strains to confirm this data, and with other assays to discard the possibility to induce chromosome aberrations. Results derived from the Comet assay discard that 33i induces DNA strand breaks and alkali-labile sites as well as possible DNA oxidation and methylation in SH-SH5Y cell line at the conditions used. Interestingly, even though a decrease in the cell survival was observed at 20 µM, it was not due to DNA damage. Thus, this is the first preliminary study confirming in vitro the lack of genotoxicity and mutagenicity induced by SIRT2 inhibition, specifically by the compound 33i.

We next assessed the effects of SIRT2 inhibition by means of the compound 33i in the APP/PS1 mouse model. The APP/PS1 mouse presents learning and memory dysfunction at 6–8 months, Aβ depositions and neuroinflammation at 6 months, increased anxiety at 12 months of age (Olensen et al. 2016) and senile plaques at 8 months (Yan et al. 2009), resembling the symptoms of early-onset familiar AD. Due to these characteristics, it is considered a suitable model to study the pathology and to investigate the potential of new therapeutic targets for AD treatment.

In agreement with recent studies using a different brain-permeable SIRT2 inhibitor (Bai et al. 2022; Wang et al. 2020; Biella et al. 2016), spatial learning, memory abilities and amyloid pathology were improved by 33i treatment in our study. Regarding other non-cognitive symptoms, we observed a decreased anxiety-like behavior in APP/PS1 mice, supporting previous studies (Reiserer et al. 2007; Lalonde et al. 2004). This symptom was not affected by 33i treatment, ruling out the involvement of any modulation of the anxious state of these animals in the cognitive improvement observed in the MWM. Noteworthy, although some authors had described an increase in anxiety in this animal model (Meng et al. 2020), it has been suggested that this is an age-related symptom that is only evident at older ages (Olensen et al. 2016). Further experiments in older animals with increased anxiety are needed to evaluate the potential anxiolytic properties of SIRT2 inhibition as has been suggested previously (Erburu et al. 2017).

Here, we also demonstrate, for the first time, that the improvement provided by SIRT2 inhibition is not only at the behavioral and molecular level, but also at the functional level since this treatment was able to reverse the impaired LTP observed in APP/PS1 mice. In addition, 33i treatment reduced the neuroinflammation in the hippocampus of APP/PS1 mice supporting previously data published in a mouse model of accelerated senescence (Diaz-Perdigon et al. 2020) and the potential of SIRT2 as a pharmacological target for the treatment of AD.

Among the mechanisms involved in the reduction of amyloid plaques, some authors have suggested that SIRT2 could have a modulatory role on APP amyloidogenic processing (Biella et al. 2016). In this sense, it has been demonstrated that SIRT2 inhibition leads to a reduction of BACE1 levels (Wang et al. 2020) and promotes APP acetylation, and therefore its non-amyloidogenic-processing (Bai et al. 2022). In the present study, we hypothesized that SIRT2 could be playing also an important role on microglial function. Increasing evidence supports the role of microglia, the cells of the brain's innate immune system, in the pathogenesis and development of AD (Gray et al. 2020). In addition, as the resident phagocytes in the CNS, microglia is responsible for identifying and eliminating pathogens (Márquez-Ropero et al. 2020). Specifically, in AD, they have been proposed to have a key role on Aβ phagocytosis and, therefore, on AD pathology progression (Park et al. 2019; Singh et al. 2022; Puntambekar et al. 2022). Interestingly, a recent study has demonstrated that SIRT2 deficiency enhances bacterial phagocytosis by macrophages (Ciarlo et al. 2017), so, we evaluated if SIRT2 inhibition could modify microglial phagocytic capacity. In agreement with the observations made in macrophages, our results demonstrate for the first time that 33i treatment increases the ability of microglia to engulf Aβ providing an additional mechanism that could contribute to the amelioration in amyloid pathology observed in 33i-treated APP/PS1 mice.

So far, the results obtained in our study supported the central beneficial effects of SIRT2 inhibition in AD; however, to date, none of the published studies regarding the potential interest of SIRT2 inhibition in neurodegenerative diseases has assessed the peripheral consequences of this treatment. Noteworthy, He and coworkers (2020) have recently shown that, while young SIRT2^−/−^ mice are metabolically normal, two-year-old SIRT2^−/−^ mice show impaired GTT and increased peripheral inflammation when compared with aged-matched WT mice. Authors hypothesize that SIRT2 is necessary for glucose homeostasis and prevents aging-related inflammation and insulin resistance (He et al. 2020). Our results obtained after three months of treatment with 33i support this notion and demonstrate that not only SIRT2 genetic ablation but also its pharmacological inhibition might have long-term adverse side effects that should not be underestimated. Indeed, although no significant differences were observed in GTT and ITT, the increase in several pro-inflammatory cytokines observed in peripheral tissues and serum samples lead us to hypothesize that longer treatments in older mice may have also negative consequences at the metabolic level and recapitulate the pathologic phenotype observed in old SIRT2^−/−^mice. Specifically, the robust increase in IL-1β observed at the periphery in 33i-treated mice support the hypothesis that SIRT2 inactivates NLRP3 inflammasome and prevents aging-associated inflammation and insulin resistance (He et al. 2020), thus its pharmacological inhibition would not be a good strategy, especially in aging. Interestingly and supporting this notion, circulating MCP-1, which is increased also after 33i treatment, has been associated to the development of insulin resistance and the increase in pro-inflammatory markers (Kamei et al. 2006).

The deleterious effect of SIRT2 inhibition at the periphery was further confirmed when the BBB impermeable SIRT2 inhibitor AGK-2 was administered. As expected, AGK-2 treatment also increased peripheral inflammatory cytokines without providing any beneficial effects at the CNS and even worsening the cognitive capacities. Therefore, future studies should focus on inhibiting SIRT2 specifically only at the brain level in order to avoid deleterious peripheral effects. This will be necessary to validate SIRT2 inhibition as a safe and effective pharmacological strategy for the treatment of AD and other neurodegenerative diseases.

The dichotomy found between central and peripheral effects of SIRT2 inhibition was also observed when we analyzed SIRT2 expression in human serum and in postmortem brain tissue samples from AD patients. In agreement with Silva et al. (2017) we observed increased expression of SIRT2 in brain cortex samples of AD patients. Interestingly, confirming these results, a recent study, with a machine-learning approach, has identified in the CSF quantifiable protein biomarkers discriminating AD from other neurological diseases and demonstrated that SIRT2 shows a very high discriminatory performance with higher CSF levels in AD patients as compared to controls (Gaetani et al. 2021). These studies support the association of SIRT2 expression in the CNS with AD pathophysiology and the interest in continuing studying the therapeutic potential of its inhibition (Sola-Sevilla and Puerta 2024). On the other hand, and in agreement with previous results (Wongchitrat et al. 2019), no significant differences were found when the expression of SIRT2 was analyzed in serum samples of AD and elderly controls. These results further support the notion that, although SIRT2 inhibition in the brain may have beneficial consequences, its pharmacological inhibition at the periphery would not be recommended.

In summary, 33i treatment was effective and beneficial in the APP/PS1 model, improving the cognitive impairment, reducing the amyloid pathology and neuroinflammation. From this perspective, considering its efficacy and the lack of mutagenicity and genotoxicity observed in the in vitro studies, this pharmacological strategy could be an ideal novel target to prevent cognitive decline and treat AD. However, supporting the increasing importance of precision medicine, our results suggest that SIRT2 inhibition is harmful at the periphery and promotes inflammation; thus, further studies are needed to maximize its therapeutic potential minimizing the possible adverse side effects.

## Material and Methods

### Drugs: SIRT2 Inhibitors

The compound 33i (2-{3-(3-fluorophenethyloxy)phenylamino}benzamide) was gently provided by Dr. Suzuki and prepared in saline with 5% dimethyl sulfoxide (DMSO; Sigma-Aldrich, Saint Louis, MO, USA) and 18% Tween 80 (Sigma-Aldrich) for in vivo experiments. For in vitro studies, 33i was dissolved in DMSO. The SIRT2-peripheral inhibitor AGK-2 (2-cyano-3-[5-(2,5-dichlorophenyl)-2-furanyl]-N-5-quinolinyl-2-propenamide) was purchased from Selleck Chemicals (Houston, TX, USA) and prepared in saline with 5% DMSO (Sigma-Aldrich) and 30% PEG-300 (Merck KGaA, Darmstadt, Germany).

### Ames Test

33i compound was assessed for mutagenicity by Ames test using TA98 *Salmonella typhimurium* strain. Four different concentrations of 33i dissolved in DMSO were tested in triplicates with and without S9 fraction. Briefly, 500 µL of PBS or 10% S9 fraction were mixed with 100 µL of bacteria (2 × 10^9^ bacteria/mL) and 50 µL of the corresponding 33i solution to obtained the concentrations to be tested (i.e., 0.5, 5, 50 and 500 µg/plate), and incubated for 1 h at 37 °C. Afterwards, 2 mL of agar containing biotin and traces of histidine was added to each mix and verted into a plaque containing solidified minimal agar medium. Positive controls were also included: 20 µg/plaque 4-Nitro-o-phenylenediamine (NPD) when no-metabolic activation was used and (con PBS) y 10 µg/plaque 2-aminofluorene (2-AF) when metabolic activation was used. Once plated, bacteria were incubated for 48 h at 37 °C and afterwards, the number of colonies observed, called revertant colonies, was counted with a laser bacterial colony counter (500A, Interscience, Saint Nom la Brétèche, France). Two independent experiments were performed.

### Cell Culture

SH-SY5Y human neuroblastoma cells were obtained from American Type Culture Collection (CRL-2266™, ATCC, VA, USA) and cultured according to standards procedures. SH-SY5Y cells were maintained in Dulbecco's Modified Eagle's Medium (DMEM; Gibco, Thermo Fisher Scientific, Waltham, MA, USA) with high glucose and supplemented with GlutaMAX™, 10% fetal bovine serum (FBS) and Penicillin–Streptomycin (10,000 U/mL; Gibco). Cells in a passage number lower than 20, were maintained in culture for no longer than 2 months since thawed. Cells were grown at 37 °C in a humidified atmosphere of 95% air / 5% CO_2_.

### Survival and Proliferation Assays

SH-SY5Y cells were seeded in 6-well plates and after 24 h, they were treated with 33i compound at 0.1–20 µM concentration range for 3 h. A negative control (i.e., cells treated with the 33i solvent -saline with 5% DMSO-) was also included. Two identical 6-well plates were seeded per independent experiment. After the treatment, cells were washed with PBS. Cells from on one of the plates, were trypsinized and counted (and used for the comet assay; see next section) using a Neubauer chamber. Fresh cell medium were added to the cells in the second plate and after 48 h were trypsinized and counted. Total suspension growth (TSG) was calculated for each condition dividing the number of cells after 48 h by the number of cells treated (before the treatment; cells plates in an extra well were trypsinized and counted just after the treatment to obtain this data). The Relative suspension growth (RSG) was calculated by dividing the TSG from each concentration tested by the TSG of the negative control. Three independent experiments were carried out.

### MTT

SH-SY5Y cells were seeded in 48-well plates and treated with 33i compound for 24 h at 37 °C. A negative control (i.e., cells treated with the 33i solvent -saline with 5% DMSO-) was also included. Afterwards, the medium was replaced by MTT reagent (0.5 mg/mL; Sigma-Aldrich) and cultures were incubated for 2 h at 37 °C. MTT was then removed, 200 µL of DMSO was added to each well and absorbance was read at 595 nm with Multiskan FC microplate reader (Thermo Fisher Sicentific).

The absorbance in the control group was assumed to represent the 100% of cell survival. The % of survival corresponding to each of the concentrations tested was calculated in relation to the negative control. Three independent experiments we performed.

### Comet Assay

The genotoxicity of the compound 33i was assessed using the standard alkaline comet assay (single-cell gel electrophoresis) and in combination with the formamidopyrimidine DNA-glycosylase (Fpg). SH-SY5Y cells were treated with different concentrations of 33i (0.1, 1, 5, 10 and 20 µM) for 3 h. A negative control (i.e., cells treated with the 33i solvent -saline with 5% DMSO-) and a positive control (i.e., cells treated with 20 µM methyl methanesulfonate (MMS) of). After the treatment cells were washed, trypsinized and counted (see previous section). Thirty 30 µL of each cellular suspension (1 × 10^6^ cells/mL) were mixed with 140 µL of 1% low melting point agarose in PBS at 37 °C. Two drops of 70 µL of the mix were placed on 1% standard agarose pre-coated slides and covered with 20 × 20 mm coverslips. Three sets of identical slides were prepared per culture called ‘Lysis’, ‘Buffer F’ and ‘Fpg’ slides. They were all immersed for 1 h in lysis solution (2.5 M NaCl, 0.1 M Na_2_EDTA, 10 mM Trizma^®^ base, 1% Triton X-100, pH 10.0) at 4 °C. Afterwards, ‘Buffer F’ and ‘Fpg’ slides were washed with Buffer F (40 mM HEPES, 0.1 M KCl, 0.5 mM Na_2_EDTA, 0.2 mg/mL BSA, pH 8.0) three times (5 min each) at 4 °C. Then, 45 µL of Buffer F or Fpg enzyme was added to each gel of their corresponding set of slides. Gels were then covered with 22 × 22 mm coverslips and incubated in a humidified chamber at 37 °C for 1 h. During this time, “Lysis” slides were kept immersed in the lysis solution at 4 °C. After that, all the slides were immersed for 40 min in alkaline solution (0.3 M NaOH, 1 mM Na_2_EDTA, pH > 13.0) at 4 °C, and then, electrophoresis was performed at 1.1 V/cm and 4 °C for 20 min. Slides were firstly neutralized with PBS and secondly with distilled water (10 min, 4 °C each wash). Finally, they were air-dried at RT.

The next day, each gel was stained with 50 µL of 1 µg/mL of DAPI solution (Sigma-Aldrich) and comets were visualized under a fluorescence microscope (Nikon Eclipse 50 i, Tokyo, Japan). DNA damage was analyzed in 100 randomly selected cells per slide (50 in each gel) by measuring tail DNA intensity (% DNA in tail) using the image analysis software Comet Assay IV (Perceptive Instruments, Cambridge, UK). The median value of the % DNA in tail was calculated for each slide. DNA strand breaks (SBs) and alkali labile sites (ALS) were measured in the “Lysis” slides, whereas Fpg-sensitive sites were calculated by subtracting the median value of % DNA in tail of the buffer F-treated slides from the Fpg-treated ones. Two independent experiments we performed.

### Animals

Experiments were carried out in male and female WT and APP/PS1 transgenic mice (5 months of age) on a C57BL/6;C3H genetic background. For microglial phagocytosis of Aβ plaques analysis, 8 months-old male and female APP/PS1 mice were used. Animals were housed in groups in standard breeding cages and had access to food and water ad libitum. Temperature and humidity were constant (23 ± 1 °C and 55 ± 10%, respectively), and lights were maintained on a 12 h light/dark cycle (light–dark: 8:00AM–8:00PM). All efforts were made to minimize animal suffering and to reduce the number of animals used in the experiments. All the procedures followed in this study and animal husbandry were conducted according to the principles of laboratory animal care as detailed in the European Communities Council Directive (2013/53/EC), are reported in compliance with the ARRIVE guidelines and were approved by the ethical committee of the University of Navarra (#051–18).

### 33i and AGK-2 Administration

WT and APP/PS1 mice were treated intraperitoneally once a day with 33i (5mg/kg) or vehicle (5% DMSO, 18% Tween 80) for 12 consecutive weeks (n = 14–16 animals per group). In a second set of experiments, the rodents were treated intraperitoneally once a day with AGK-2 (5mg/kg) or vehicle (5% DMSO, 30% PEG-300) for 12 consecutive weeks (n = 5–8 animals per group). Behavioral tests were carried out at 8^th^ week of treatment. Sample size for the studies were chosen following previous studies in our laboratory (Diaz-Perdigon et al. 2020) and using one of the available interactive web sites (http://www.biomath.info/power/index.html).

### Elevated Plus Maze Test

In order to take into account any possible effect of the 33i treatment on the anxious state of the animals, elevated plus maze (EPM) was performed. It is an elevated platform with two close and two open arms, crossed in the center oppositely one to another with a middle region. Mice were placed inside one of the arms and were permitted to move freely between them for 5 min. The frequency to enter in the open arms and the total time spent in them were used as a measure of an anxiety-like behavior.

All trials were monitored by a video camera set above the mazes and connected to a video tracking system (Ethovision XT 5.0, Noldus, Wageningen, The Netherlands).

### Morris Water Maze

The Morris Water Maze (MWM), a hippocampus-dependent learning task used to analyze the spatial memory and to assess the working and reference memory, was performed as previously described (Sola-Sevilla et al. 2021) with minor modifications. The water maze consisted of a circular pool (diameter of 145 cm) filled with water (21–22 °C) and virtually divided into four equal quadrants (northeast, northwest, southeast, and southwest). To guide the mice, visual cues were placed in the room.

Firstly, mice underwent visible-platform training for 2 days (6 trials per day), in which a platform was located in the southwest quadrant raised above the water with an object placed on top to facilitate its location (Habituation phase). In this phase, it was confirmed that all mice exhibited a normal swimming pattern and were able to reach the platform.

For assessing learning capacity (Acquisition phase), a hidden platform (1 cm below the water surface) was placed in the northeast quadrant of the pool. The trial was finished when the animal reached the platform (escape latency) or after 60 s in the pool. After each trial, mice remained on the platform for 15 s. The test was conducted over 7 consecutive days (4 trials per day). In both Habituation and Acquisition phases, mice were placed pseudo-randomly from different locations in each trial, facing towards the wall of the pool to eliminate the potentially confounding contribution of extra maze spatial cues. Moreover, the order of the entry positions varied each day.

To test memory retention, the platform was removed, and animals were allowed to swim for 60 s (Retention phase). This trial was performed on days 5^th^ and 8^th^ (last day) of the test and the percentage of time spent in the northeast quadrant was recorded.

All trials were monitored by a video camera set above the center of the pool and connected to a video tracking system (Ethovision XT 5.0, Noldus).

### Electrophysiology

Synaptic transmission in hippocampal slices of vehicle and 33i treated WT and APP/PS1 mice was analysed as previously described (Zamora-Moratalla and Martín 2021). Briefly, transverse brain slices of 400 μm thick were cut with a vibratome and incubated for at least 1 h at RT in artificial cerebrospinal fluid (aCSF) gassed with a 95% O_2_/5% CO_2_ mixture at pH 7.3–7.4. Individual slices were then transferred to an immersion recording chamber and perfused with oxygenated warmed aCSF (32 ± 2 °C). Field postsynaptic potentials (fEPSPs) were recorded in the stratum radiatum of the CA1 pyramidal layer by a carbon fiber microelectrode (Carbostar-1, Kation Scientific, Minneapolis, MN, USA). Evoked fEPSPs were elicited by stimulation of the Schaffer collateral fibers with an extracellular bipolar tungsten electrode placed in the stratum radiatum. At the beginning of each experiment, basal synaptic transmission was analyzed by applying isolating stimuli of increasing intensity to reach a maximal fEPSP response. For Long-term potentiation (LTP) experiments, the stimulus intensity was adjusted to elicit 50% of the maximum response signal and kept constant throughout the experiment. After recording stable baseline responses for 30 min, LTP was induced by a single train of theta burst stimulation (TBS; 5 bursts of 5 pulses at 100 Hz, with an interval of 200 ms between bursts). Potentiation was measured for 1 h after LTP induction at 0.033 Hz.

### Glucose- and Insulin-Tolerance Tests

In Glucose-Tolerance Test (GTT), mice were fasted for 6 h with free access to water. Animals were intraperitoneally administered with 20% of D-glucose (Merck KGaA). Blood glucose concentrations were measured before (baseline) and after 15, 30, 60 and 120 min of glucose administration by venous tail puncture using glucometer and *Accu-Check Aviva* glucose strips (Hoffmann-La Roche, Basel, Switzerland).

For Insulin-Tolerance Test (ITT), animals were fasted for 1 h with free access to water. After basal glucose in blood was determined (baseline), insulin was intraperitoneally administered (0.75 UI per kg of body weight; Actrapid, Novo Nordisk, Bagsværd, Denmark) and glycaemia was measured after 15, 30 and 60 min using a glucometer and *Accu-Check Aviva* glucose strips (Hoffmann-La Roche).

### Analysis of Microglial Phagocytosis of Aβ Plaques

In vivo Aβ phagocytosis was determined following a flow cytometry-based protocol described by Lau et al. (2021) with some modifications. 8 month-old APP/PS1 mice were treated intraperitoneally once a day with 33i (5mg/kg) or vehicle (5% DMSO, 18% Tween 80) for 1 week (n = 6 animals per group). The 8t^h^ day, methoxy-X04 (MeX04) was injected intraperitoneally (10mg/kg; Tocris Bioscience, Bristol, UK). After 3 h, mice were perfused transcardially with ice-cold PBS under xylazine/ketamine anesthesia. Hippocampus and cortex were collected, chopped into pieces and digested together with papain (0.4 mg/mL; Worthington Biochemical Corporation, Lakewood, NJ, USA) in a 37 °C water bath with shaking for 30 min. Then, the samples were mechanically dissociated, and the cellular suspension was filtered through a 70 μm cup Filcon cell suspension filter (BD, Franklin Lakes, NJ, USA) into a solution of 20% FBS/80% HBSS (Gibco). After a centrifugation of 200 g for 5 min, the pellets were resuspended in 5 mL of 80% HBSS/20% Percoll solution. After creating an interphase with HBSS, samples were centrifuged at 200 g for 20 min. This interphase was removed, and cells were centrifuged at 4500 rpm for 5min. Cells were washed and incubated with anti-mouse CD45 (1:100; eBioscience, Thermo Fisher Scientific) and anti-mouse CD11b (1:200; BioLegend, PerkinElmer, Waltham, MA, US) monoclonal antibodies for 30 min on ice. After washing the cells, they were resuspended in 350 µL of sorting buffer (0.5% bovine serum albumin or BSA, 2.5mM EDTA in PBS). Samples were analyzed in a FACS Canto II flow cytometer (BD) and using *FlowJo* software (BD). For analysis, the CD11b^+^CD45^low^ population was gated. WT mice injected with methoxy-X04 were used to determine the methoxy-X04-threshold for non-phagocytosing cells, and unstained wild-type cells were used to determine background fluorescence.

### RNA Extraction and Quantitative PCR

Total RNA was isolated from hippocampus and epididymal white adipose (Epi-WAT) samples using *TRI Reagent*^*®*^ (Sigma-Aldrich). One microgram of RNA was retrotranscribed into cDNA using the *High-Capacity cDNA Reverse Transcription Kit* (Applied Biosystems, Waltham, MA, USA), and quantitative real-time PCR was carried out on CFX384 Touch™ Real-Time PCR Detection System (Bio-Rad, Hercules, CA, USA) using *iQTM SYBR*^*®*^* Green Supermix* reagent (Bio-Rad). Primers used are detailed in the following table (Table 1). For hippocampus samples, *Gapdh* was used as an internal control, whereas *36b4* gene expression was used for Epi-WAT samples.

**Table 1:** Primers sequences used for SYBR Green qPCR analysis

	**Forward primer (5’- 3’)**	**Reverse primer (5’- 3’)**
***Il-1β***	TGAAATGCCACCTTTTGACA	AGCTTCTCCACAGCCACAAT
***Il-6***	GTTCTCTGGGAAATCGTGGA	TCCAGTTTGGTAGCATCCATC
***Tnf-α***	TGCCTATGTCTCAGCCTCTT	TGATGAGAGGGAGGCCATTT
***Tgf-β***	TTGCTTCAGCTCCACAGAGA	TGGTTGTAGAGGGCAAGGAC
***Gapdh***	CCAAGGTCATCCATGACAAC	TGTCATACCAGGAAATGAGC
***36b4***	AACATCTCCCCCTTCTCCTT	GAAGGCCTTGACCTTTTCAG

For *Abca1* determination, quantitative real-time PCR was carried out using *Taqman*^*®*^* Universal PCR Master Mix* (Applied Biosystems) and ViiA™ 7 Real-Time PCR System (Applied Biosystems). Both *Abca1* and *Gapdh* (internal control) primers were from Applied Biosystems (cat# Mm00442646_m1 and Mm99999915_g1, respectively).

For gene expression quantification, the double delta CT (ΔΔCT) method was used where delta CT (ΔCT) values represent normalized target genes levels with respect the internal control (*Gapdh* or *36b4*). The relative quantification of all targets was carried out using the comparative cycle threshold method, 2^−ΔΔCt^, where ΔΔCt = (Ct target gene − Ct endogenous control) treated/(Ct target gene − Ct endogenous control) untreated.

### Western Blot

For Western blot analysis, hippocampal and Epi-WAT tissues were sonicated in cold lysis buffer with protease inhibitors (0.2 M NaCl, 0.1 M HEPES, 10% glycerol, 200 mM NaF, 2 mM Na_4_P_2_O_7_, 5 mM EDTA, 1 mM EGTA, 2 mM DTT, 0.5 mM PMSF, 1 mM Na_3_VO_4_, 1 mM benzamidine, 10 mg/mL leupeptin, 400 U/mL aprotinin) and incubated on ice for 30 min. After centrifugation at 13,000 rpm for 20 min, the supernatant was collected. In the case of Epi-WAT samples, the upper fat layer was also removed.

In order to measure total protein concentration, Bio-Rad protein assay was performed, following the manufacturer’s protocol (Bio-Rad). Equal amounts of protein (30 μg for hippocampal tissues, and 20 μg for Epi-WAT) were separated by electrophoresis on a sodium dodecyl sulphate–polyacrylamide gel (7.5%) under reducing conditions and transferred onto a nitrocellulose membrane (Hybond-ECl; Amersham Bioscience, Amersham, UK) for 16 h at 4 °C. The trans-blots were blocked in TBS-Tween containing 5% powder milk for 1 h. Membranes were probed overnight at 4 °C with rabbit polyclonal antibody anti-GluA1 (1:1000; cat# AB1504, Sigma-Aldrich). As internal control, mouse monoclonal anti-β-Actin was used (1:1000; cat# A1978, Sigma-Aldrich).

The next day, membranes were incubated with goat polyclonal anti-rabbit (cat# 926–68021) and anti-mouse (cat# 926–32210) secondary antibodies (1:5000; Odyssey, LI-COR Biosciences, Lincoln, NE, USA) for 2 h at RT. Bands were visualized using Odyssey Infrared Imaging System (LICOR Biosciences). Results were calculated as the optical density values of WT-Vehicle mice.

### Immunofluorescence

Brains of six mice per experimental group were histologically processed for Aβ plaques and Iba1 determination. After dissection, one brain hemisphere was postfixed for 24 h with 4% paraformaldehyde and conserved in 30% sucrose for 1 week. Serial coronal brain slices (thickness: 40 μm) were cut with a freezing microtome and stored in cryoprotectant solution.

Free-floating slices were washed 3 times in PBS and incubated in 70% formic acid for 10 min to expose the Aβ epitope. Then, brain sections were incubated in blocking solution (PBS containing 0.5% Triton X-100, 0.1% BSA, and 2% normal donkey serum) for 2 h at RT. Afterwards, slices were incubated with mouse monoclonal anti-β-amyloid (1:200; cat# 803001, BioLegend) and rabbit polyclonal anti-Iba1 (1:1000; cat# 019–19741, Fujifilm Wako, Osaka, Japan) primary antibodies overnight at 4 °C. Sections were washed with PBS and incubated with the secondary antibody Alexa Fluor donkey anti-mouse 488 (1:200) and Alexa Fluor goat anti-rabbit 568 (1:250) for 2 h at RT, protected from light (cat# A-21202 and A-11011, respectively; Thermo Fisher Sicentific). Finally, sections were washed with PBS and mounted with *DAPI Fluoromount-G*^*®*^* Mounting Medium* (Southern Biotech, Birmingham, AL, USA).

In order to ensure comparable immunostaining, sections were processed together under same conditions. Images were acquired with the Vectra Polaris scanner (Perkin Elmer). Quantification of fluorescent signal in brain sample images was carried out using *ImageJ* program (NIH, Bethesda, MD, USA).

### Quantification of Aβ-42 Levels in Brain Cortex

For Aβ-42 levels quantification, 20 mg of brain cortex were homogenized in 8 volumes of cold 5 M guanidine-HCl in 50 mM Tris buffer. The homogenate was incubated for 3 h at RT on an orbital shaker and then, it was diluted ten-fold with cold PBS supplemented with 1X protease inhibitor cocktail (Calbiochem, Merck KGaA). Samples were centrifuged 20 min at 16,000 g at 4 °C and the supernatant was diluted 1:2000 with Standard Diluent Buffer provided with the ELISA kit. Fifty microliters of the resultant solution were assayed using the *Ultrasensitive Amyloid-β 42 Human ELISA Kit* (cat# KHB3544; Invitrogen) following the manufacturer’s instructions. Each sample was analyzed in duplicate.

### Quantification of IL-1β In Epididymal White Adipose Tissues

25 mg of Epi-WAT from each animal was sonicated in *Cell Lysis Buffer 2* (cat# 895347; R&D systems, Minneapolis, MN, USA) at 1:5 dilution and incubated on ice for 30 min. Samples were then centrifuged 12 min at 13,000 rpm at 4 °C and fifty microliters of the resultant supernatant were assayed in the *Mouse IL-1 beta/IL-1F2 Quantikine ELISA Kit* (cat# MLB00C; R&D systems) following the manufacturer’s protocol.

### Quantification of Cytokines in Serum

For the quantitative measurement of cytokines in serum samples, *BD Cytometric Bead Array (CBA) Mouse Inflammation Kit* (cat# 552364; BD) was used. Interleukin-6 (IL-6), Interleukin-10 (IL-10), Monocyte Chemoattractant Protein-1 (MCP-1), Interferon-γ (IFN-γ), Tumor Necrosis Factor (TNF), and Interleukin-12p70 (IL-12p70) protein levels were assayed following the manufacturer’s instructions. Briefly, fifty microliters of serum were incubated with Capture Beads and Mouse Inflammation PE Detection Reagent for 2 h at RT, protected from light. After washing the samples, data acquisition was performed with a *FACS Canto II* flow cytometer (BD) and analyzed using *FlowJo* software (BD).

### SIRT2 Expression in Postmortem Brain Tissue Samples from AD Patients

Brain tissues were obtained from the *Oxford Project to Investigate Memory and Ageing* (OPTIMA, see www.medsci.ox.ac.uk/optima). Subjects for this study constituted a randomly selected subset of the participants, now part of the Thomas Willis Oxford Brain Collection within the Brains for Dementia Research Initiative (BDR). At death, informed consent had been obtained from the patients’ next-of-kin before collection of brains and the study was approved by the UK National Research Ethics Service. All cases were selected based on clinic-pathological consensus diagnoses. Participants classified as normal controls (n = 10), did not have dementia or other neurological diseases, did not meet CERAD criteria for AD diagnosis, and were staged at Braak 0-II. AD cases (n = 10) were clinically diagnosed based on meeting the *Consortium to Establish a Registry for Alzheimer’s Disease* (CERAD) criteria for a diagnosis of probable or definite AD. Frontal (Brodmann Area, BA10) cortex were dissected free of meninges. In the control group, the average age was 69.8 years (SD 3.06) and the sex distribution was 6/4 men/women. In the AD group, the average age was 80.4 years (SD 2.12) and sex distribution was 3/7 men/women. All tissue used had a brain pH > 6.1, condition used as an indication of tissue quality in post-mortem research.

mRNA extraction from 20 mg of human brain tissue was performed using the *Nucleospin RNA kit* (Macherey–Nagel, Düren, Germany) according to the manufacturer's instructions. Afterwards, 500 ng of RNA was retrotranscribed into cDNA using the *High-Capacity cDNA Reverse Transcription Kit* (Applied Biosystems), and quantitative real-time PCR was carried out using Taqman^®^ Universal PCR Master Mix (Applied Biosystems) and ViiA™ 7 Real-Time PCR System (Applied Biosystems). Both *SIRT2* and *β-ACTIN* (internal control) primers were from Applied Biosystems (cat# Hs01560289_m1 and Hs01060665_g1, respectively).

For Western blot analysis, 10 mg of human brain tissue were sonicated in cold lysis buffer with protease inhibitors (0.2 M NaCl, 0.1 M HEPES, 10% glycerol, 200 mM NaF, 2 mM Na_4_P_2_O_7_, 5 mM EDTA, 1 mM EGTA, 2 mM DTT, 0.5 mM PMSF, 1 mM Na_3_VO_4_, 1 mM benzamidine, 10 mg/mL leupeptin, 400 U/mL aprotinin) and incubated on ice for 30 min. After centrifugation at 13,000 rpm for 20 min, the supernatant was collected. Equal amounts of protein (50 μg per sample) were separated by electrophoresis on a sodium dodecyl sulphate–polyacrylamide gel (7.5%) under reducing conditions and transferred onto a nitrocellulose membrane (Hybond-ECl; Amersham Bioscience, Amersham, UK) for 16 h at 4 °C. The trans-blots were blocked in TBS-Tween containing 5% powder milk for 1 h. Membranes were probed overnight at 4 °C with rabbit polyclonal antibody anti-SIRT2 (1:1000; cat# S8447, Sigma-Aldrich). The next day, membranes were incubated with goat polyclonal anti-rabbit (cat# 926–68021) secondary antibody (1:5000; Odyssey, LI-COR Biosciences, Lincoln, NE, USA) for 2 h at RT. Bands were visualized using Odyssey Infrared Imaging System (LICOR Biosciences). As internal control, mouse monoclonal anti-β-Actin was used (1:1000; cat# A1978, Sigma-Aldrich).

### Quantification Of SIRT2 in Human Serum Samples

Human serum samples were obtained from the Karolinska University Hospital Memory Clinic in Huddinge (Sweden) including control (n = 26) and AD (n = 28) patients, for a total of 54 samples. The average age was 68 years (SD 9.42), and the sex distribution was 23/77% men/women (n = 6/20) in the control group, and 36/64% men/women (n = 10/18) in the AD group. The clinical and diagnostic data of the GEDOC cohort are described in detail in Goikolea et al. (2022) and Rosenberg et al. (2019).

For the quantitative measurement of SIRT2 protein in human serum samples from control and AD patients, *Human SIRT2 SimpleStep ELISA Kit* (cat# ab227895; Abcam, Cambridge, UK) was used. Serum samples were 2X-diluted in the assay diluent buffer. Briefly, fifty microliters of diluted serum samples were incubated with the Antibody Cocktail for 1 h at RT on a plate shaker. Next, samples were incubated with TMB Development Solution, the reaction was stopped after 10 min and the OD was recorded at 450 nm. The concentration of SIRT2 protein was determined by interpolating the sample absorbance values (blank subtracted) against the standard curve and multiplying by the dilution factor.

### Statistical Analysis

In vitro experiments were analyzed by one-way ANOVA followed by Dunnett’s multiple comparison test. In the habituation and acquisition phase of the MWM, body weight, GTT and ITT, strain and treatment effects were analysed by repeated-measures two-way ANOVA followed by multiple comparisons with Tukey’s test. The rest of the behavioral tests and biochemical results were analyzed using two-way ANOVA (strain*treatment) followed by multiple comparisons with Tukey’s test was used. Post hoc test was applied only if F on interaction was significant. In figure legends, the F values represent the F of interaction followed by the p-value of the corresponding post hoc test. In those cases where the F of interaction was not statistically significant, the F value shown represents the main effect observed strain or treatment. Microglial Phagocytosis of Aβ plaques, Aβ-42 levels and Aβ plaques quantification as well as SIRT2 analysis in human samples were analyzed by unpaired parametric Student’s t test.

Results were expressed as mean ± standard error of the mean (SEM), and differences among groups were considered statistically significant at p < 0.05. All the statistics were performed by *GraphPad Prism* software (San Diego, CA, USA).

### Supplementary Information

Below is the link to the electronic supplementary material.

Supplementary file1 (DOCX 160 KB)

## Data Availability

The data presented in this study are available on request from the corresponding author.
