# Supplementary material for: SIRT2 Inhibition Rescues Neurodegenerative Pathology but Increases Systemic Inflammation in a Transgenic Mouse Model of Alzheimer’s Disease
Source: J Neuroimmune Pharmacol. 2023 Sep 12;18(3):529–50. doi: 10.1007/s11481-023-10084-9 (PMC10577113; doi:10.1007/s11481-023-10084-9)
Supplement: Supplementary file 1 — Supplementary file1 (DOCX 160 KB) [file 11481_2023_10084_MOESM1_ESM.docx]

**SUPPLEMENTAL MATERIAL**


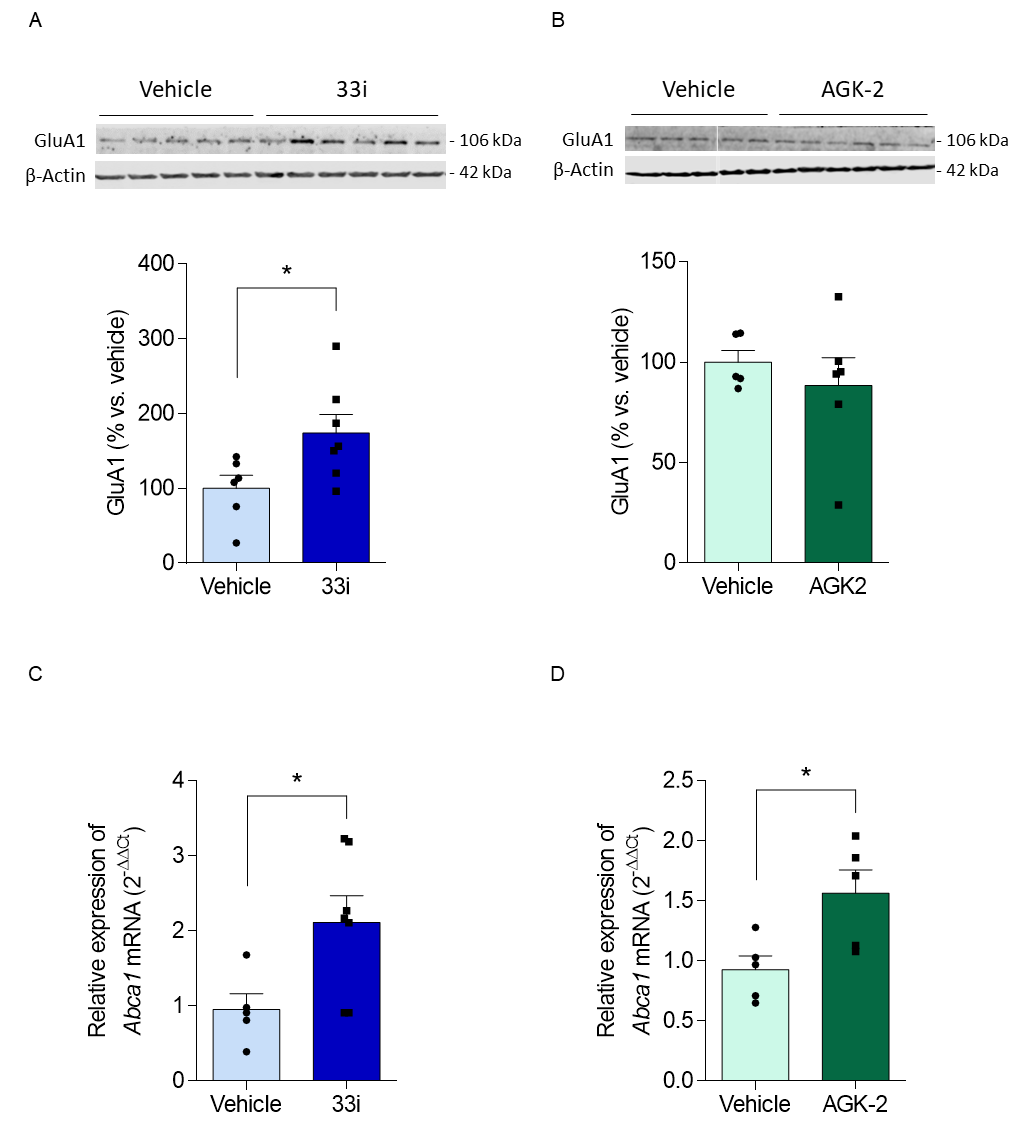


**Fig. S1 Pharmacological inhibition of SIRT2 by compounds 33i and AGK-2 at central and peripheral levels.** Hippocampal GLUA1 expression after 33i (**a**) or AGK-2 treatment (**b**). Note that only 33i treatment increases significantly the expression of GlUA1 protein in the hippocampus, confirming the inability of AGK-2 to cross the BBB (**p* < 0.05 Student’s t test). β-actin was used as internal and loading control. Peripheral gene expression of *Abca1* after 33i (**c**) or AGK-2 (**d**) treatment. The inhibitory effect of both compounds on the SIRT2 enzyme at the periphery is evidenced by an increase in the expression of *Abca1* in white adipose tissue. Results are shown as mean ± SEM (n = 5-7 animals per group).

**
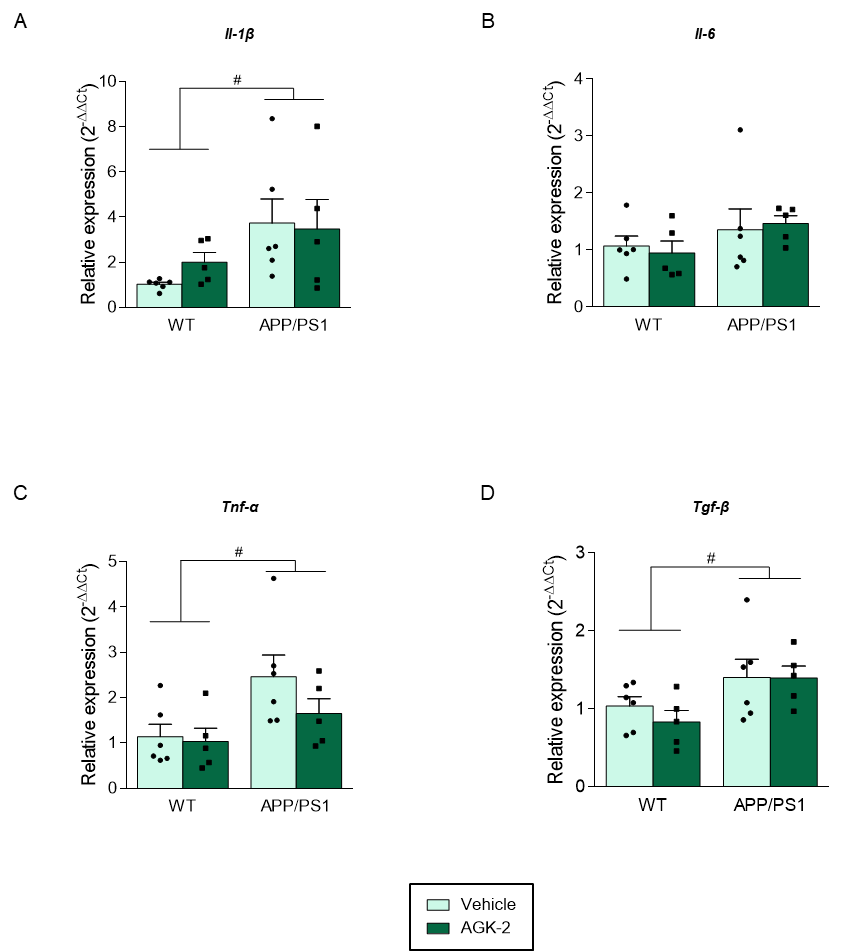
**

**Fig. S2 AGK-2 treatment does not reduce neuroinflammation in APP/PS1 mice.** Hippocampal gene expression of (**A**) *Il-1β* (*F* = 5.968, ^#^*p* < 0.05, main effect of genotype), (**B**) *Il-6,* (**C**) *Tnf-α* (*F* = 7.108, ^#^*p* < 0.05, main effect of genotype) and (**D**) *Tgf-*$\beta$ (*F* = 7.079, ^#^*p* < 0.05, main effect of genotype). Note that no significant differences are observed between vehicle or AGK-2 treated animals. *Gapdh* was used as an internal control. Results are shown as mean ± SEM (two-way ANOVA, n = 5-6 animals/group)
